# User Perspectives of Geriatric German Patients on Smart Sensor Technology in Healthcare

**DOI:** 10.3390/s23229124

**Published:** 2023-11-11

**Authors:** Marcin Orzechowski, Tobias Skuban-Eiseler, Anna Ajlani, Ulrich Lindemann, Jochen Klenk, Florian Steger

**Affiliations:** 1 Institute of the History, Philosophy and Ethics of Medicine, Ulm University, 89081 Ulm, Germany; tobias.skuban-eiseler@uni-ulm.de (T.S.-E.); anna.ajlani@jku.at (A.A.); florian.steger@uni-ulm.de (F.S.); 2 Department of Sociology with a Focus on Innovation and Digitalization, Institute of Sociology, Johannes Kepler University Linz, 4040 Linz, Austria; 3 Department of Geriatrics, Robert Bosch Hospital, 70376 Stuttgart, Germany; ulrich.lindemann@rbk.de (U.L.); jochen.klenk@rbk.de (J.K.); 4 Institute of Epidemiology and Medical Biometry, Ulm University, 89081 Ulm, Germany; 5 Department of Health Sciences and Healthcare Education, IB University of Health and Social Sciences, Study Center Stuttgart, 70178 Stuttgart, Germany

**Keywords:** wearable sensors, smart sensors, older adults, rehabilitation, healthcare, ethics

## Abstract

With consideration of the progressing aging of our societies, the introduction of smart sensor technology can contribute to the improvement of healthcare for older patients and to reductions of the costs of care. From the clinical and medico-ethical points of view, the advantages of smart sensor technology are copious. However, any ethical evaluation of an introduction of a new technology in medical practice requires an inclusion of patients’ perspectives and their assessments. We have conducted qualitative, semi-structured, exploratory interviews with 11 older patients in order to gain their subjective opinions on the use of smart sensor devices for rehabilitation purposes. The interviews were analyzed using methods of qualitative content and thematic analyses. In our analysis, we have focused on ethical aspects of adoption of this technology in clinical practice. Most of the interviewees expressed their trust in this technology, foremost because of its accuracy. Several respondents stated apprehension that the use of smart sensors will lead to a change in the patient–healthcare professional relationship. Regarding costs of introduction of smart sensors into healthcare, interviewees were divided between health insurance bearing the costs and individual participation in corresponding costs. Most interviewees had no concerns about the protection of their privacy or personal information. Considering these results, improvement of users’ technology literacy regarding possible threats connected with putting smart sensors into clinical practice is a precondition to any individual application of smart sensors. This should occur in the form of extended and well-designed patient information adapted to individual levels of understanding. Moreover, application of smart sensors needs to be accompanied with careful anamnesis of patient’s needs, life goals, capabilities, and concerns.

## 1. Introduction

The progressing aging of the population constitutes one of the major challenges for healthcare systems in numerous countries. The demographic shift of societies is mostly visible in high-income societies; however, this trend will also increasingly affect low- and middle-income countries. According to predictions of the United Nations World Health Organization, by the year 2050, 80% of older people will be living in these countries [1]. More than 15% of the world’s population will be over 60 years old by the year 2030, and the number of older people by this time will increase to 1.4 billion from 1 billion in 2020. The United States Census Bureau reports that in 2020, the number of older people reached 55.8 million, or 16.8% of the population, and grew nearly five times faster than the total population over the last 100 years [2]. The number of older persons already surpassed the 20% benchmark in several European countries: Germany, Bulgaria, Croatia, France, Latvia (22%); Finland, Portugal, Greece (23%); Italy (24%) [3]. It currently reaches 30% in Japan [3].

The provision of high-quality healthcare for aging patients increasingly becomes a challenge for overburdened, understaffed, and underfinanced healthcare systems [4,5]. With augmenting demand for healthcare services, the provision of timely and quality medical care increasingly depends on technological support. For example, the use of artificial intelligence and machine learning in diagnosis and therapy has been suggested as one of the possible solutions for the question of allocation of scarce resources in healthcare [6,7].

Similarly, introduction and dissemination of sensors and wearable technology contributes to the improvement of patients’ care. Sensors in healthcare are unobtrusive, small, and light wearable devices developed for measurement and gathering of a patient’s individual data [8]. Combined with machine learning systems, so-called smart sensors communicate gathered data to a sensing network, record, and analyze it [9]. Therefore, smart sensors constitute valuable support systems for clinical decision making in a multitude of clinical application areas, e.g., for monitoring and analyzing ECG patterns in cardiovascular diseases [10], monitoring blood glucose concentration in patients suffering from diabetes mellitus, or in renal, respiratory, and tumor diseases [8,9,11]. Above these, smart sensors have been used for rehabilitation purposes, e.g., in postoperative rehabilitation. For this purpose, wearable sensors are placed on the body of patients. These sensors record and transmit acceleration in space as inertial data, which is then analyzed and assigned to complex movement patterns [12]. Up to now, existing rehabilitation programs mostly rely on patients’ subjective reports of their rehabilitation activities conducted in an outpatient setting, which carries the risks of lower adherence or false data reports [13]. Use of smart sensor technology can minimize these risks and allow physicians to objectively assess the data, evaluate the treatment progress, and adapt the future rehabilitation measures [14]. In order to record and analyze activity patterns in geriatric rehabilitation, smart sensors in the form of activity trackers can be worn on the body in the living environment of the patients. This makes it possible to analyze the radius of action of patients, to detect falls, and to examine the frequency of everyday activities.

The advantages of smart sensors have been amply shown in the literature. They allow patients to be more individually involved in monitoring, planning, and conduct of healthcare activities, which leads to a reduction of healthcare costs and an improvement of the quality of health services [15]. From the ethical point of view, the use of smart sensors contributes to a shift from paternalistic to more cooperative and patient-centered healthcare, in which patients are more involved in their own care through increased self-monitoring and self-management of their illness [16]. This has positive effects on patients’ knowledge of their health status, their health behavior, and general autonomy [17]. In the case of rehabilitation of older patients, smart sensors allow for monitoring at home, thus granting patients the possibility of maintaining their daily routines without interruptions caused by monitoring in a hospital setting [18]. However, the use of smart sensors for rehabilitation purposes depends heavily on patient compliance [19]. Patients’ decisions about participation in rehabilitation programs in which smart sensors are applied may be influenced by numerous factors [20]: patients may lack confidence in technology and thus be anxious that their medical care depends on decisions made by a machine, not by a healthcare professional. Moreover, patients may have concerns about protection of their privacy, as daily measurements conducted by sensors mean intrusion in their activities, and collected data may be accessed by third parties. Furthermore, an obstacle from the point of view of the patients may be costs of this technology that need to be carried by the patients themselves. Additionally, especially in the case of older patients, low technology literacy, i.e., low knowledge of handling modern digital devices, may lead to distrust or even opposition to using them. Therefore, any ethical evaluation of the introduction of smart sensors into rehabilitation of patients requires an inclusion of patients’ perspectives and their assessments. Although research on this topic has progressed in recent years, inclusion of wider, patient-centered perspectives is required to support adoption of this technology not only from the clinical but also from the medico-ethical point of view. In order to complement this research gap, we designed and conducted qualitative interviews with potential users of smart sensors designed for rehabilitation of geriatric patients; that is, devices that can detect, gather, and analyze movement patterns or falls. Consequently, the aim of our investigation presented in this paper was to inquire potential users of smart sensors in rehabilitation about their subjective opinions on risk and benefits connected with this technology.

## 2. Materials and Methods

### 2.1. Participants and Ethics

In order to explore the patients’ perspectives, problem-centered, semi-structured interviews were conducted with older patients requiring rehabilitation in a south-west German hospital making them potential candidates for an application of smart sensors. Inclusion criteria were an age of 65 years or older, a sufficient cognitive status, and possible movement limitations in their daily routine. Exclusion criteria were insufficient German language skills and terminal illness. Persons who expressed willingness to participate in interviews received detailed information about the research project, its aims, procedures of protection of their personal data, and the possibility of withdrawing their consent for participation at any time. Only persons who expressed their consent were invited for an interview. The study protocol was approved by the ethical committee of the medical faculty at the University of Tuebingen (1009/2020BO2; 3 February 2021), and all participants gave written informed consent.

### 2.2. Interviews

The purpose of the qualitative interviews was to gain insight into subjective opinions of the interview partners on the use of smart sensor devices for their rehabilitation purposes. The method of qualitative, semi-structured, exploratory interviews is a valuable tool to achieve this aim and is one of the major research methods in applied ethics. First, it allows for collection of individual perspectives of the respondents, their previous experiences, or motives for certain actions [21,22]. Second, it provides a certain flexibility in the conduct of the interviews; while prepared questions and interview guidelines allow the interviewer to focus the inquiry on issues central to the investigation, the possibility to ask ad hoc questions grants an opportunity to elucidate interviewees’ statements or to clarify issues raised by the interviewees [23,24].

Preparation of the interview questions was based on extensive research and ethical analysis of the scientific literature on the topic [19,20]. Central ethical issues ensuing from this previous investigation were formulated in the form of questions and interview guidelines. The focus of the interviews was to query the participants of the interviews about their opinions on smart sensor technology using activity tracking as an example.

The interviews were conducted in person, in the native language of the participants by a male senior researcher with substantial knowledge of the research topic and its practical context. Each of the interviews was based on the same catalogue of questions. The interviews were not structured according to topics. Since the research questions do not specifically aim at analysis of the influence of individual characteristics of interviewees, i.e., age, gender, different levels of education, severity of diseases requiring rehabilitation, or financial capabilities, on the research topic, no demographic data have been collected during the interviews.

The interviews were digitally recorded and subsequently transcribed. After transcription, the interviews were fully anonymized. Written transcriptions served as a basis for analysis of the content of the interviews. This analysis followed the procedure of qualitative content analysis [24,25] and thematic analysis [26,27]. Three researchers (M.O., A.A., T.S-E.) separately analyzed the transcripts and derived main themes mentioned by the interview partners. The analysis followed the sequential phases for thematic analysis: (i) familiarization with the data, (ii) generation of initial codes, (iii) search for the themes, (iv) review of the themes, (v) definition of the themes, and (vi) reporting the results [27]. During the process of the analysis, the responses were reduced to their core elements, manually coded, extracted, and systemized through clustering into main themes and subthemes. These themes mirror important recurring topics touched upon during the interviews. The results of the separate analyses were compared and discussed with regard to the occurring differences. The transcripts of the interviews were analyzed in the language of the interview. In order to illustrate reported topics, representative quotes from the interviews were translated from German into English.

## 3. Results

### 3.1. Participants

Twelve interviews were conducted, of which eleven could be analyzed due to withdrawal of consent for the research by one participant. The issue of conducting further interviews was then discussed in the research team with the conclusion that satisfactory data saturation was achieved, and no further interviews were necessary. The age of the 11 interview partners ranged between 73 and 90 years (median 77). Six interviewees identified themselves as female and five as male. Furthermore, all participants reported mobility limitations for in- and outdoor activities.

### 3.2. Topics

**Figure 1:**
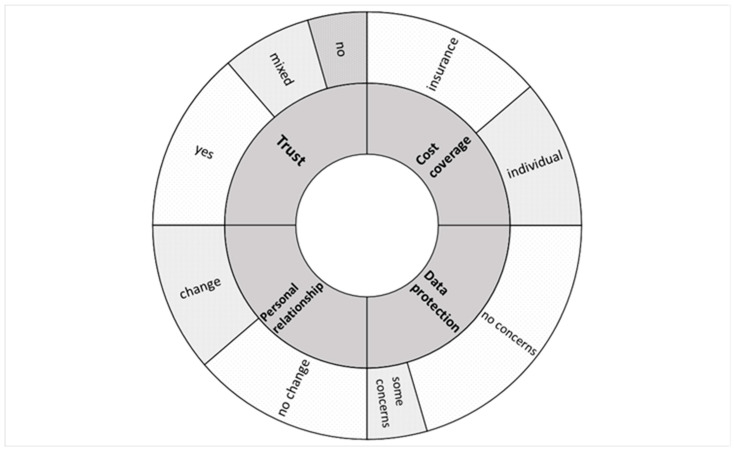
Graphic representation of four main topics (ring 1) touched upon by interview partners and their opinions on these topics (ring 2). Size of the area of each field in ring 2 represents proportion of the opinions on the relevant topic.

The analysis of the interviews showed that four major topics emerged during the talk with interview partners: (i) issues of trust towards the use of smart sensors in healthcare; (ii) challenges for the patient–healthcare professional relationship in a scenario in which smart sensors are used for rehabilitation purposes; (iii) protection of patients’ privacy and data gathered by smart sensors; (iv) issues of cost coverage for use of this technology for individual rehabilitation of the patients. Graphic representation of main themes that arose in the interviews and interviewees’ responses is provided in Figure 1.

#### 3.2.1. Trust toward Smart Sensors Technology

The first of the main themes that emerged in the interviews was the question of trust towards the measurements and analyses conducted by smart sensors and the recommendations provided by them. Generally, most of the interview partners (N = 6) expressed their trust in this technology, foremost because of its accuracy.

*“Oh well, I assume that they [devices] can measure more meticulously”*

(iv 3)

Moreover, smart sensors were considered more objective than healthcare professionals, who can often be distracted or under time pressure. As one of the interviewees presented it:

*“As a rule, people [do] always what suits them at the time. And that’s why I prefer the machine.”*

(iv 7)

*“The treatment team needs a foundation on which to build [the recommendation]. And that is, let’s call it, objective. And the moment it’s [done] with the person [involved], it’s never objective, it’s always subjective. And that’s just the reason why I trust the machine more as an assistance.”*

(iv 6)

Respondents in this group were of the opinion that if a technology is authorized for use in medical areas, it must be proofed as accurate and safe.

However, several interview partners (N = 3) opted rather for the use of smart sensors as support for clinical recommendations, which should effectively be taken by healthcare professionals. Here, cooperation between a professional and a device was deemed as advantageous. Trust towards a device or an algorithm is possible in such a case if it is substantiated by human expertise.

*“Well, basically, the machine measurements are more objective, but I’m currently considering whether a mixture could possibly be an option. That means that machine measurements are always carried out and, in case of doubt, in certain areas they are perhaps substantiated again by people or (…) questioned.”*

(iv 2)

*“The machine detection of movements can be very precise. The individual process (…) by the examiner can also be justified. The sensors are there and [they are] recording and, yes, I can’t influence the interpretation, but I assume that it won’t be done lightly.”*

(iv 5)

Only two interview partners responded that they would not trust the measurements or recommendations made by smart sensors. A personal connection to the patients and better accuracy of the attending person were mentioned as the reasons against solely relying on digital devices.

*“[Because of] human connection.”*

(iv 8)

*“Well, because she [healthcare professional] notices the mistakes while she is working and can correct them.”*

(iv 1)

#### 3.2.2. Relationship with the Attending Healthcare Professionals

The relationship and communication with the attending medical team arose as another focal point for the interviewees. Here, the opinions of our respondents were divided; while N = 5 of them predicted that the application of smart sensors will lead to a change in the relationship between patients and healthcare professionals, N = 6 were of the opposite opinion.

Among the respondents who expected a change in the relationship, two interviewees assumed that objectivity and precision of automated activity tracking will be a useful addition to diagnostic procedures and will improve the existing situation of care.

*“[If] I don’t know whether I should move, how much I [should] move, what kind of movements [should] I make (…), I think, it would be good [that the sensor would] give [me] advice on (…) movements.”*

(iv 10)

*“If questions arise here or if difficulties arise here, well, basically I would trust the smart controls (…). If problems or if different views or opinions arise, the question (…) is whether one is trying to achieve a unified opinion by simply discussing the points. So, if the person being examined has different opinions about the smart results, then maybe you should talk to [healthcare professional] who knows the system better.”*

(iv 2)

However, the introduction of algorithmic decision support systems was also deemed a concern for disruption of familiar interactions. The concerns were expressed in two areas: loss of personal care and reduced involvement in therapeutic decision making. One interview partner replied to the question whether he expected a change in the relationship with their team of providers quite affirmatively:

*“Because the doctor then only has to look at this printed data and no longer has to speak to me. And then there is the danger that one [healthcare professional] generally trusts an algorithm more than perhaps a conversation with the patient. And the algorithm doesn’t always have to be right.”*

(iv 3)

For another interviewee, the negative side of predicted change was a loss of personal contact with the attending healthcare professionals, who will mostly deal with the data gathered by the sensors and neglect the personal situation of the patient:

*“The personal contact [between the patient and the attending team] will simply be missing. (…) Or it will be reduced.”*

(iv 8)

#### 3.2.3. Data and Privacy Protection

When asked about their considerations regarding data and privacy protection, most interviewees (N = 9) answered that they had no concerns in relation to handling their activity data or personal information by the clinic staff.

*“In principle no. I can’t imagine anyone having any special interest in it.”*

(iv 5)

*“You don’t even know my private sphere. And we’re only together for a few minutes, when so many people [patients] are here, you have less contact [with individual one], right?”*

(iv 7)

However, two of the interview partners responded that, while they do not have any concerns regarding handling their personal and activity data by the clinic staff, they expressed some concerns about who else can have access to this data. In both cases, the interviewees mentioned insurance companies, which can have particular interest in the data gathered by smart sensors from patients, especially if they cover the cost of the devices.

*“(…) I assume that they [insurance companies] are interested in this data.”*

(iv 3)

*“The cost coverage by the health insurance companies means that the health insurance companies also have the right to access this data.”*

(iv 5)

#### 3.2.4. Cost Coverage of the Use of Smart Sensors

The fourth notable main topic in the interviewees’ responses was the question of cost coverage for the application of smart sensors technology in rehabilitation. Due to necessary hardware acquisitions and new requirements for the clinical infrastructure, the implementation of sensor-based activity tracking can cause a short-term increase of expenses. When asked whether they would prefer a full reimbursement through health insurance or if they would consider acquiring a sensor-based rehabilitation treatment through individual coverage, the respondents expressed divided opinions. Six interviewees gravitated towards public health insurance coverage. This preference was reasoned for with mentions of precarious pensions and an expected benefit through early prevention and personalized therapeutic measures for insurers. Here, an individual financial situation played a major role. One interviewee elaborated on having to prioritize financial stability over personalized therapeutic measures.

*“[Sensor-based activity tracking is] for health insurance and in retirement, you’re not exactly wealthy and we used to say, every penny, now every cent, is important to me. And everything that goes off the pension money is draining my wallet.”*

(iv 6)

*“The problem is that I can’t cover the high costs. Well, I’d be willing to pay a small fee, but I can’t afford to pay a lot.”*

(iv 11)

Another interviewee conveyed that smart sensors may not have individual benefit for him right now, and future development of this technology will bring advantages for other patients and insurance companies.

*“(…) it may not even be in my interest. Maybe it does not lie in my interest and still I have to use it.”*

(iv 3)

One interview partner valued the involvement of insurance companies as an additional control mechanism or indicator of quality and necessity since the involvement of health insurance meant to them that there was *“(…) someone else auditing”* (iv 12).

On the other side, five interviewees expressed willingness to participate financially in the cost, or at least a part of it, of use of smart sensors. In this group, the respondents mostly focused on individual benefits that introduction of this technology may bring to them.

*“Yes, if I have something out of it, I would also bear the costs.”*

(iv 1)

*“If the matter is important to me, for my personal situation, I would participate [in the costs] because it is in my own interest.”*

(iv 2)

*“I would participate [in the costs] if it would be beneficial to my health, so to speak, or to monitoring my health (…).”*

(iv 8)

*“A small contribution [to the costs], I could imagine. Because I also have an advantage from it and also get information. And I also have an advantage when my doctor has information, because it can flow into the therapy or the physiotherapy. Because I’m the beneficiary.”*

(iv 10)

## 4. Discussion

The aim of this study was to investigate the perspectives of older patients who represent a target group for the use of smart sensors in rehabilitation. We wanted to identify their subjective opinion regarding the risks and benefits of this technology in order to provide an essential prerequisite for a differentiated ethical analysis of the application of this modern technology in rehabilitation. Older patients have been asked about their attitudes to different sensor monitoring systems in the past [28,29,30,31]. Our results are in line with the findings that older patients generally have a high acceptance of sensor monitoring systems in their home environment [28,29,30,31]. Obviously, the use of new technology in the home environment does not seem to be strongly problematized by older patients. Our results confirm this finding. However, a difference was found in the attitude towards data security, which will be discussed later.

We were able to recruit interview partners who would represent a suitable target group for smart sensors in rehabilitation. Interviewees were of older age, the gender ratio was balanced, and activities reported by study participants to be problematic showed significant movement limitations.

In terms of the trust that study participants expressed in smart sensors, the majority communicated high trust in the technology. The interview partners justified this by stating that they would rate smart sensors as more objective than healthcare professionals, who would often be under time pressure and distracted. Only two interviewees said they did not have confidence in the technology, and three interviewees advocated that smart sensors could be an appropriate adjunct to support health professionals’ assessments and recommendations. The trust that the majority of interview partners express in the technology finds a counterpart in studies on the use of clinical decision systems supported by artificial intelligence in geriatrics. Here, too, the positive aspects associated with the technology are emphasized above all [32]. At the same time, the older persons are a clientele that generally has a rather low level of technology literacy [33,34,35]. Therefore, the question arises as to what effect an increase in technology literacy would have on the interviewees’ assessment of their confidence in the new technology. However, it is becoming increasingly difficult to provide sufficient information that older people can understand regarding new technologies in healthcare, as they become more and more complex [36]. There is a high need to provide adequate and sufficient information to older people when using modern technologies so that they are able to develop sufficiently well-founded trust in the technology or not.

In terms of challenges regarding the patient–healthcare professional relationship, about half of the interviewees were of the opinion that the use of smart sensors may have an impact, while the other half disagreed. In terms of potential changes to the relationship, the following concerns were expressed: the use of technology could lead to a loss of human contact in healthcare, and there could also be a loss of patient involvement in therapeutic decisions. These concerns have also been expressed in the literature regarding other modern technology in geriatrics [37]. Maintaining direct face-to-face healthcare professional–patient communication must not be jeopardized by the use of modern technology under any circumstances. It is important to remember that personal communication with health professionals has a strong impact on the physical and mental health of older patients. It not only involves the exchange of medical information, but also involves emotional and affective care, which significantly contributes to the health care of older patients [38]. Patients are very different in terms of their needs and what is and is not possible for them in their individual life situation. For older people in particular, it is important to carefully weigh up personal restrictions against individual wishes and needs. This is only possible if adequate personal contact with the older patients continues to be maintained. The “capability approach” [39], which is significant in ethical research in geriatrics [40,41], points out that it is not a question of resources that determines whether older people can achieve their personal goals [42], but that individual wishes and needs and a patient’s own possibilities must be carefully weighed against each other [19]. Otherwise, respect for patient autonomy, which is considered highly significant in medical ethics, could be at risk. If respect for patient autonomy implies that conditions must be created that make such respect possible in the first place [43], then modern technologies such as smart sensors can be part of such conditions if their use does not jeopardize contact with patients.

Nine interviewees had no concerns about privacy and data protection when using smart sensors, and only a minority of two interviewees expressed concern that third parties might have access to the collected data. In particular, our interviewees were concerned that health insurers would have access to the data collected because they pay for the cost of the technology. The relatively low level of data security concerns identified in our study is initially surprising. Interestingly, concerns about data security when using modern technologies in geriatrics are also rarely mentioned in corresponding studies [32]. This is surprising, because data security issues are highly relevant and storing patient data may well potentially lead to harm for patients—for example, if these data are accessed by unauthorized third parties [44]. This could involve cyberattacks by which personal data of older patients might be withdrawn [45]. The assumed low technology literacy could contribute to the fact that older patients are not even aware of or underestimate the dangers that could be associated with modern technologies. This was clearly visible in the response of one of our interviewees (iv 5), who stated that he cannot imagine that anyone has any interest in his personal data.

This implies the high ethical desideratum that those responsible ensure at all times that the data of older patients are handled well, that they cannot be accessed by third parties, and that they are also deleted after an appropriate period of time. The results on the attitude of older patients towards data security were very different in past studies. The collection of private data was considered unproblematic when the positive effects on daily life outweighed the data security issues [29]. Other studies found both older patients’ concerns about data security [30] and willingness to share personal data [31]. One study showed that various concerns of older patients about such modern technologies decrease as older patients gain more experience with the technologies [28]. These findings in combination with our results could hint at the fact that older patients may need more information about new technologies and an appropriate handling of them in order to be able to develop resilient and clear attitudes.

Regarding the assumption of costs for the modern technology, about half of all interviewees were in favor of public health insurance bearing these costs, while the other half were willing to participate in the corresponding costs themselves. Those who would not contribute to the costs argued that older people had little money to spend anyway. Furthermore, they pointed out that the use of smart sensors could ultimately prove positive for health insurance companies: smart sensors would make a contribution to prevention, hereby saving subsequent health costs. The interviewees who were willing to contribute to the costs argued with the personal advantages that such a technology could have for them. Both lines of reasoning might apply. However, through them, argumentative reference is made in each case to different important medical principles. According to Beauchamp and Childress [46], beneficence and justice are two of the four principles that must be strongly considered in medical ethics reflections. The two principles are not mutually exclusive, yet a different weighting of beneficence or justice may result in individuals being willing or unwilling to share in the costs of modern technologies. Apparently, some interviewees rated social justice higher than their own well-being and vice versa. This could be related to the fact that some interviewees would expect greater benefits from the use of technology in relation to themselves and others less. To our knowledge, the question of why some individuals are willing to pay for the use of modern technologies in healthcare and others has not yet been sufficiently explored. It is possible that factors such as personal wealth, education level and an awareness of one’s own health are important to consider here [47]. The use of smart sensors can contribute to social justice in medicine, as it may succeed in providing better and more effective care to a higher number of elderly patients.

It should not be forgotten to ask the older patients themselves about their attitudes and fears regarding the use of modern technologies in healthcare, in order to be able to evaluate the technologies ethically as comprehensively as possible. In order not to overlook older patients’ needs, it remains of high necessity to involve them in the development of new health technologies and the implementation of these technologies in healthcare [48]. That Patient and Public Involvement and Engagement can improve the quality of research is not only true in the case of older patients and is increasingly recognized [49]. This work would like to contribute to engaging older patients in the development of new technological devices in healthcare.

## 5. Limitations

The results of this research must be regarded in light of its limitations. The small sample of interviewed individuals from a limited geographical location in Germany does not allow for generalization of the results in global or even national perspective. However, generalization of results is not the purpose of qualitative research in the field of medical ethics. The aim is rather to present a subjective view from a particular group of stakeholders. This can contribute to a better understanding of their personal positions, concerns, and perspectives. Moreover, the size of the sample allows a detailed inspection of interviewees’ responses. Such an approach grants the possibility to make visible personal attitudes towards the topics of the research that are otherwise often overlooked in research conducted with the use of other, e.g., statistical, methods. Therefore, in order to substantiate the results of this research, they require triangulation in further investigations. We conducted interviews with 11 participants. For qualitative interview studies, this is actually not a small sample set. Typically, one identifies most of the data in qualitative interviews in a few initial interviews [50,51]. In most cases, it is already the first six interviews that provide the essential data [50].

A further limitation of the research is a lack of individual information on the background of the interviewees. However, we have not collected or analyzed demographic data or specific information on the background of interviewees as for the purpose of this study we do not analyze correlation or influence of these factors on interviewees’ opinions presented in the paper. The aim of qualitative interviews conducted by us is to gain insights into the subjective ethical views of the interviewees on the issues being the topic of the research. Correlations between subjects’ individual background and their views on the topic of the study may be an interesting point of further research, which, however, should be conducted with the use of other research methods.

Furthermore, the subjectivity of the coding system is a limitation of the method of thematic analysis. Moreover, only the occurrence of certain themes is identified in the analysis with the use of this method—the relationship between the themes remains outside the scope of the analysis. Nevertheless, thematic analysis has the advantage of allowing for identification of various aspects pointed out in the interviews, which can be a starting point for further research.

## 6. Conclusions

The central result of our research is that older patients seem not to have considerable reservations regarding the use of smart sensors in their clinical rehabilitation. However, this must be interpreted against the background of a presumably low level of technology literacy of the interviewees, which should be considered as a challenge in implementation and use of these devices in geriatric context. Thus, the interviewees did not consider data security issues to be an obstacle and expressed high trust in the new technology. Apparently, they are not fully aware of possible threats that careless use of the technology might bring along. Improvement of their knowledge regarding possible pitfalls connected with putting smart sensors into clinical practice might be a precondition to any individual application of smart sensors. This should occur in the form of extended and well-designed patient information that is adapted to an individual level of understanding.

With regard to the quality of the individual patient–healthcare professional relationship, interviewees expressed concerns despite a possible low level of technology literacy. In the introduction of smart sensor technology, caring and empathic relations between older patients and health professionals must not be disrupted at any time.

Taking into account the “capability approach” [39], which is very important in geriatric research [40,41], the application of smart sensors in rehabilitation needs to be accompanied with careful specific anamnesis of patient’s needs, life goals, capabilities, and concerns. To this aim, support in the form of ethical consultation regarding individual patients can be favorable and effective.

The influences of higher technology literacy on patient assessment of the use of smart sensors in rehabilitation should be better researched in the future. It would be informative to know whether patients’ basic trust in technology changes with higher knowledge. This could be a suitable argumentation basis for spending more time and effort in educative measures for older patients when introducing new technologies in their care.

## Data Availability

The data presented in this study are available on request from the corresponding author. The data are not publicly available due to privacy protection reasons.
